# Numbers, neighbors, and hungry predators: What makes chemically defended aposematic prey susceptible to predation?

**DOI:** 10.1002/ece3.6956

**Published:** 2020-11-24

**Authors:** Jan M. Kaczmarek, Mikołaj Kaczmarski, Jan Mazurkiewicz, Janusz Kloskowski

**Affiliations:** ^1^ Department of Zoology Poznań University of Life Sciences Poznań Poland; ^2^ Department of Inland Fisheries and Aquaculture Poznań University of Life Sciences Poznań Poland

**Keywords:** amphibian, aposematism, associational effects, mimicry, predator hunger, tadpole

## Abstract

Many chemically defended aposematic species are characterized by relatively low toxin levels, which enables predators to include them in their diets under certain circumstances. Knowledge of the conditions governing the survival of such prey animals—especially in the context of the co‐occurrence of similar but undefended prey, which may result in mimicry‐like interactions—is crucial for understanding the initial evolution of aposematism. In a one‐month outdoor experiment using fish (the common carp *Cyprinus carpio*) as predators, we examined the survival of moderately defended aposematic tadpole prey (the European common toad *Bufo bufo*) with varying absolute densities in single‐species prey systems or varying relative densities in two‐species prey systems containing morphologically similar but undefended prey (the European common frog *Rana temporaria*). The density effects were investigated in conjunction with the hunger levels of the predator, which were manipulated by means of the addition of alternative (nontadpole) food. The survival of the *B. bufo* tadpoles was promoted by increasing their absolute density in the single‐species prey systems, increasing their relative density in the two‐species prey systems, and providing ample alternative food for the predator. Hungry predators eliminated all *R. temporaria* individuals regardless of their proportion in the prey community; in treatments with ample alternative food, high relative *B. bufo* density supported *R. temporaria* survival. The results demonstrated that moderately defended prey did benefit from high population densities (both absolute and relative), even under long‐term predation pressure. However, the physiological state of the predator was a crucial factor in the survival of moderately defended prey. While the availability of alternative prey in general should promote the spread and maintenance of aposematism, the results indicated that the resemblance between the co‐occurring defended and undefended prey may impose mortality costs on the defended model species, even in the absence of actual mimicry.

## Graphical abstract

In a one‐month outdoor experiment using fish as predators, we examined the survival of moderately defended aposematic toad tadpole prey with varying absolute densities in single‐species prey systems or varying relative densities in two‐species prey systems that contained morphologically similar but undefended prey (frog tadpoles). The density effects were investigated together with the hunger levels of the predator. The survival of the toad tadpoles was promoted by increasing both their absolute density and their relative density, and by providing ample alternative food for the predator. The results demonstrated that moderately defended prey do benefit from their high numbers. However, the resemblance between the co‐occurring defended and undefended prey species, even in the absence of actual mimicry, as well as the physiological state of the predator, may impose significant mortality costs.

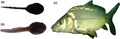

## 1 INTRODUCTION

Being chemically defended and aposematic does not make prey fully immune to predation. Predators show varying levels of innate avoidance of aposematic prey (Brodie & Brodie, 1999; Guilford, 1990; Lindström et al., 1999), and they often must learn to avoid such targets (Exnerová et al., 2007; Svádová et al., 2009). Additionally, many prey species are only moderately defended; they are unpalatable to predators, but are not severely toxic, which enables the predators to include them in their diets (Skelhorn et al., 2016). The decision of the predator to attack or ignore such prey is influenced by an array of factors, including both intrinsic, such as the present or past nutritional state (Bloxham et al., 2014), previous experience with the prey (Exnerová et al., 2007), learning ability (Rowland et al., 2017), and toxin burden (Rowland et al., 2010) and extrinsic, such as the abundance of defended prey (Lindström et al., 2001), availability of alternative resources (Carle & Rowe, 2014), variation in the toxicity (Gamberale‐Stille & Guilford, 2004) and nutrient content of the prey (Halpin et al., 2014), and even ambient temperature (Chatelain et al., 2013).

Investigating the survival of defended prey under varying conditions is essential for understanding the evolution of aposematic signaling (Halpin et al., 2017; Riipi et al., 2001; Ruxton et al., 2004). The survival of aposematic prey is usually higher when they are present in the environment at high densities and/or in aggregations (Gamberale & Tullberg, 1996, 1998; Hotová Svádová et al., 2014; Rowland et al., 2013). This increase in survival is because predators that encounter the prey frequently should learn about their defenses more quickly (Fisher, 1930; Greenwood et al., 1989; Riipi et al., 2001). Additionally, the predator may also quickly reach the limits of its ability to metabolize ingested toxins (Turner & Speed, 1999). Importantly, defensive grouping may provide additional antipredator benefits that are not associated with chemical defenses, such as dilution effects (reviewed in Lehtonen & Jaatinen, 2016). The abundance of alternative food resources for the predator is usually beneficial for the defended prey, which facilitates the evolution of defenses and/or aposematic signaling (Lindström et al., 2004; Mappes et al., 2005; Sherratt et al., 2004). This occurs because hungry predators exhibit decreased selectivity, while well‐nourished predators generally refrain from attacking any less profitable prey (Hileman et al., 1994; Kokko et al., 2003; Sandre et al., 2010). Nevertheless, in some contexts, the presence of alternative prey may lead to heightened mortality rates in defended prey (“associational susceptibility”; Underwood et al., 2014). For example, if the defended prey are restricted to a habitat patch, the presence of edible neighbors may encourage the predator to concentrate its feeding effort in the area, with the defended prey suffering collateral damage (“apparent competition”; Barbosa et al., 2009; Holt & Kotler, 1987). In another context, if the harmless prey resemble the aposematic prey, especially if they imitate the warning signal (Batesian mimicry), their presence may interfere with the learning process of the predator, leading to reduced survival of the defended model species (Jones et al., 2013; Lindström et al., 1997). The learning process can be hindered even when the undefended prey are poor or imperfect mimics (Kikuchi & Pfennig, 2013; Sherratt, 2002).

We investigated the survival of moderately chemically defended aposematic prey (hereafter defended prey) that were subject to predation in response to their varying absolute densities in single‐species prey communities. We also investigated the survival of the aposematic prey in response to their varying relative densities in two‐species prey communities containing undefended prey potentially capable of causing associational susceptibility (hereafter: undefended prey). In all cases, the treatments were also manipulated by varying the nutritional state of the predator. Specifically, we investigated how the survival of tadpoles of the European common toad *Bufo bufo* L. (defended prey) under predation pressure from the common carp *Cyprinus carpio* L. was affected by (a) the absolute density of the *B. bufo* tadpoles in the single‐species prey systems (Experiment 1); (b) the relative density of the *B. bufo* tadpoles in the two‐species prey systems with tadpoles of the European common frog *Rana temporaria* L. (undefended prey) where the absolute density of *B. bufo* was kept constant while the density of *R. temporaria* was changed across the treatments (Experiment 2); and (c) the physiological state of the fish predator (the hunger level, which was manipulated by varying additions of alternative nontadpole food in both experiments). Previously, we demonstrated the presence of associational interactions between *B. bufo* and *R. temporaria* tadpoles under favorable conditions (high levels of alternative nontadpole food for the predator). In that case, *R. temporaria* exhibited increased mean individual survival when co‐occurring at low relative density with *B. bufo* (“associational resistance”; Kaczmarek et al., 2018). The duration of our experiments encompassed almost the entire free‐swimming phase of the tadpoles of the prey species to obtain the realistic cumulative effects of predation on the survival rates.

We predicted the following effects: (a) In the single‐species prey systems, increased absolute density of defended prey would translate to higher survival rates (Experiment 1); (b) in the two‐species prey systems, decreased relative density of defended prey would translate into lower survival rates for the defended prey, that is, the occurrence of associational susceptibility (Experiment 2); and (c) increased hunger of the predator should lead to reduced survival of all prey regardless of defenses (Experiments 1 and 2).

## 2 MATERIALS AND METHODS

**Figure 1:**
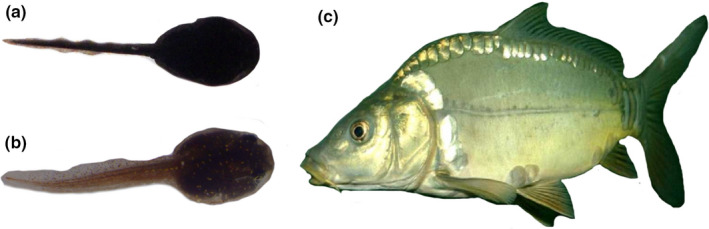
Species used in the study: (a) common toad *Bufo bufo* (early tadpole stage, chemically defended prey); (b) common frog *Rana temporaria* (early tadpole stage, undefended prey); (c) common carp *Cyprinus carpio* (predator). The images are not to scale

The European common toad *B. bufo*, widespread in Europe and Western Asia (Garcia‐Porta et al., 2012), usually breeds in deep ponds inhabited by fish (Van Buskirk, 2003). Its tadpoles are gregarious and black, a color considered aposematic in larval anurans (Wells, 2007). The skin of both tadpoles and adults contains alkaloids and toxic protein compounds (Kowalski et al., 2018), rendering them unpalatable to vertebrate predators, including fish (Glandt, 1984; Üveges et al., 2019). The European common frog *R. temporaria* is a habitat generalist (Van Buskirk, 2005), widely distributed across Europe (Sillero et al., 2014). The tadpoles of *R. temporaria* lack chemical defenses and are cryptically colored (Glandt, 1983). Tadpoles of the two species exhibit morphological differences (Van Buskirk, 2002), but share the generalized pond tadpole morphology (cf. Petranka & Kennedy, 1999). They react differently to fish predator cues: *R. temporaria* exhibits behavioral avoidance of fish and marked reduction in swimming activity, whereas *B. bufo* shows a weak or no response (Laurila, 2000; Nyström & Åbjörnsson, 2000). Tadpoles of *R. temporaria* are also known to exhibit induced morphological changes in the presence of predators (Stamper et al., 2009; Van Buskirk, 2001, 2002). Adults of *R. temporaria* do not necessarily avoid oviposition in ponds containing fish (Indermaur et al., 2010; Laurila & Aho, 1997; Van Buskirk, 2005); however, their reproductive success is limited by the presence of fish (Bardsley & Beebee, 1998; Laurila, 1998). Although generally exhibiting different preferences for reproductive habitats, the two species are regularly found together in breeding habitats (Gazzola & Van Buskirk, 2015; Indermaur et al., 2010) and their breeding phenologies overlap (Sparks et al., 2007). Taken together, these features make them potential candidates for the emergence of associational effects. The common carp *C. carpio* is a large omnivorous fish, originally distributed from southeastern Europe to eastern Asia, but with a much broader range at present due to introductions (Balon, 1995). This carp, although not a specialized predator of mobile nektonic macrofauna, readily forages on tadpoles when they are available (Kloskowski, 2011). Species used in the experiment are shown in Figure 1.

The experiments were conducted in concrete ponds (6 × 6 m, adjustable water depth set at 75 cm) at the Muchocin experimental station of the Poznań University of Life Sciences (52°37′14.47″N; 15°50′36.84″E) from 2016 to 2018. The experimental setup consisted of mesh enclosures (100 × 100 × 100 cm, submerged up to 75 cm) with cube‐shaped steel frames for internal support, placed in the ponds. The ponds, with four evenly distributed enclosures in each, were individually supplied with water from the neighboring river (nutrient range values: 0.25–1.02 mg PO_4_^−^ L^−1^; 0.1–0.5 mg NO_3_^−^ L^−1^; total nitrogen 2.16–7.37 mg N/L) that passed through inlets fitted with fine‐mesh screens. The walls of the enclosure (1 mm mesh size) were inserted into the bottom of the basin, giving the tadpoles and fishfree access to the sandy substrate. A submerged PVC mesh cylinder (100 cm in length, 10 cm in diameter, 10 mm mesh size) was fastened to the bottom in each enclosure to create habitat complexity and to provide a refuge structure for tadpoles. The enclosures were covered with PVC mesh (10 mm mesh size) on the top to prevent predatory insects from entering. All enclosures were inoculated with zooplankton by adding 7 L of natural pond water.

The study investigated the changes in the survival of chemically defended *B. bufo* tadpoles and in their fitness‐related traits (mass at metamorphosis) along a gradient of tadpole density (both absolute and relative), crossed with two levels of availability of alternative food for the predators, with some replication across the gradient. The study consisted of two experiments. In both experiments, the response variables were survival of *B. bufo* tadpoles to metamorphosis and their mass at metamorphosis. In Experiment 1, *B. bufo* tadpole survival in single‐species prey communities with varying absolute density was tested in the presence of a fish predator. Two independent variables were manipulated: *B. bufo* tadpole density (5/30/40/50/80 individuals per enclosure) and the level of alternative nontadpole food (feed pellets) provided for the fish. Availability of alternative food was considered as a proxy for hunger level in the predators; “high food” (100 g twice a week) versus “low food” (2 g twice a week). In Experiment 2, *B. bufo* tadpole survival in two‐species prey communities with *R. temporaria* was tested in the presence of a fish predator, with manipulation of the relative (but not absolute) density of *B. bufo*. The density of *B. bufo* tadpoles was kept constant (30 individuals/enclosure), while *R. temporaria* tadpole density varied (5/10/30/50/60 individuals per enclosure), as did the level of alternative, nontadpole food provided for the fish (same as in Experiment 1). In both experiments, each enclosure contained one 1‐year‐old specimen of *C. carpio* (total length 100–130 mm) as a predator. The experimental design did not include predator‐free treatments; this was based on our previous result, i.e., that the mortality of tadpoles at Gosner stage 25 or above was low in the absence of predation (Kaczmarek et al., 2018). The tadpole densities used in the experiment were selected so as not to exceed the mean values reported for natural ponds (Gazzola & Van Buskirk, 2015; Loman, 2004). However, *B. bufo* densities in single‐species treatments were occasionally higher, as tadpoles of this species often form large and dense aggregations (Watt et al., 1997); this was intended to reflect densities within aggregations rather than on the scale of a whole pond.

The tadpoles used in the experiment originated from amplexed pairs of *B. bufo* and *R. temporaria* collected from ponds in Wielkopolska province (NW Poland) and housed for approximately 1 week in separate pens, 1 m^3^, partially submerged in water. The obtained egg masses and hatched larvae remained there until *B. bufo* tadpoles reached Gosner stage 25. At this stage, the tadpoles were collected (tadpoles of *R. temporaria* were slightly larger than those of *B. bufo* as a result of hatching earlier in the season), randomly assigned to treatments, and stocked into experimental enclosures. All anurans used in the study were later returned to their original habitats. The fish used in the experiment originated from semi‐natural ponds and had no experience with anuran tadpoles as prey. For one week prior to the experiment, the fish were kept indoors in large fiberglass tanks and provided ad libitum with commercial fish feed (pellets: 35% total protein, 9% crude lipid) that was also later used during the experiment. Individual fish were placed singly in experimental enclosures one day after the enclosures were stocked with tadpoles. Apart from the feed, the fish could also forage on the natural invertebrate prey present in the substrate and in the water column. The mean increases in total fish body length in the low‐ and high‐food treatments during the experiment were measured in 2018, equaling 18% and 31%, respectively. The enclosures were stocked in early May of each year (range: 5–12 May), and the metamorphs were collected after 4–5 weeks. The emerging metamorphs (Gosner stage 46) were collected by dip netting and were counted and weighed to the nearest 0.01 g. The individuals that failed to complete metamorphosis during the experiment were included in the analysis of survival but not that of body mass.

The study was conducted over three consecutive years, in uniform environmental conditions. During each year of the research, Experiment 1 and Experiment 2 were conducted in different ponds. Numbers of replicates were uneven between treatments and seasons. Most replicates were performed in 2017 and 2018; four replicates for each experiment were carried out in 2016. Moreover, in 2017 and 2018, marsh frogs *Pelophylax ridibundus* oviposited in several of the ponds and, as some spawn remained undetected, the freshly hatched tadpoles were able to intrude, entering the enclosures through the mesh walls; the invaded enclosures were excluded from the data analysis. However, we believe that the unbalanced design of the experiments should not greatly affect the results; since we were investigating tadpole survival along density gradients and not across categorical levels (except for the manipulation of alternative nontadpole food availability for fish predators), we were more interested in obtaining a broad gradient of different densities or proportions of defended/undefended tadpoles than in complete replication of all treatment combinations. The final analyses were performed on a set of 17 (Experiment 1) and 48 (Experiment 2) enclosures. The details on the final allocation of treatments and sample sizes are presented in Table 1.

**Table 1:** Summary of the experimental design

Experiment	Initial number of *B. bufo* tadpoles	Initial number of *R. temporaria* tadpoles	Alternative nontadpole food level	Number of replicates			
				2016	2017	2018	Total
Experiment 1 (single‐species prey system with *B. bufo*)	5	—	Low		2		2
			High				—
	30	—	Low		3		3
			High				—
	40	—	Low				—
			High	4			4
	50	—	Low		2	1	3
			High			2	2
	80	—	Low		1	1	2
			High			1	1
Experiment 2 (two‐species prey system)	30	—	Low		2		2
			High				—
	30	5	Low		4	2	6
			High			2	2
	30	10	Low		4	2	6
			High	4		3	7
	30	30	Low		4	2	6
			High			4	4
	30	50	Low		4	2	6
			High			4	4
	30	60	Low			1	1
			High			4	4

Mixed models were applied throughout to control for nonindependence of data (e.g., the use of multiple enclosures per pond) and to compensate for the unbalanced dataset. Variance components were estimated by the restricted maximum‐likelihood (REML) procedure; REML is better suited for the analysis of unbalanced data than conventional analysis of variance (Patterson & Thompson, 1971). Survival of *B. bufo* tadpoles to metamorphosis was compared between treatments using generalized linear mixed models (GLMMs) with a logit link and binomial distribution. The number of survivors was treated as a binomial response; the initial number of *B. bufo* tadpoles constituted the binomial denominator. In all models, the availability of alternative nontadpole food (low vs. high food) was entered as a fixed factor. The other fixed terms, which were treated as continuous variables, were the initial number of *B. bufo* tadpoles per enclosure in the models of the single‐species system (*B. bufo* only) and the initial relative density of *B. bufo* in the tadpole community in the two‐species prey system (with *R. temporaria*). The initial relative density was expressed as the proportion of *B. bufo* tadpoles in the initial total number of tadpoles. Since replicates within ponds and years were not entirely independent, the pond and year were entered as random factors. In the two‐species prey systems, we also evaluated the survival of *R. temporaria* to metamorphosis in relation to the relative density of *B. bufo* in the tadpole community. However, survival was modeled only for the high‐food treatments, since no *R. temporaria* metamorphs emerged from the enclosures when low levels of nontadpole food were available to fish (see Section 3).

The factors affecting *B. bufo* mass at metamorphosis were analyzed using a GLMM with a normal distribution and an identity‐link function. The model contained the same set of fixed and random terms as the respective survivorship models, but the enclosure variable was nested within the pond variable as a random factor to account for the lack of independence within the enclosures. To adjust for potential effects of population thinning on mass at metamorphosis, final *B. bufo* density was entered in the analysis of the two‐species prey system but not of the single‐species prey system, since the densities of surviving *B. bufo* metamorphs were strongly intercorrelated with both the initial tadpole densities and the availability of alternative food for fish.

Interactions between the level of alternative food for fish and *B. bufo* density (proportion) were initially included in the models, but removed where nonsignificant to increase model stability; for tadpole survival, models with interactions did not converge. The analyses were performed in GenStat 15.0 (VSN, Hemel Hempstead, UK).

## 3 RESULTS

**Figure 2:**
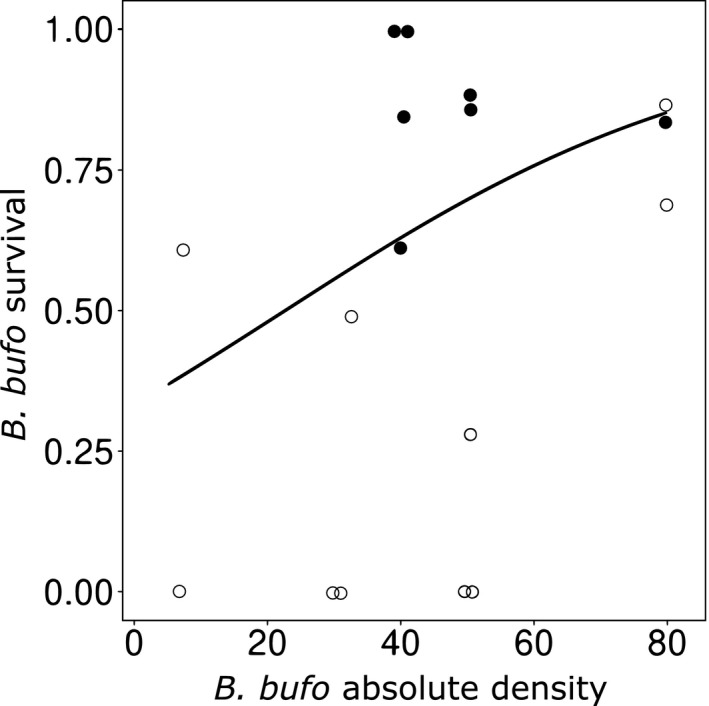
Survival to metamorphosis of moderately chemically defended *B. bufo* tadpoles along the gradient of the initial absolute densities of conspecifics in experimental enclosures with fish predators. Data points are individual replicates. Circles indicate enclosures with low (empty circles) or high (filled circles) levels of alternative non‐tadpole food (fish feed pellets) for the predator. The overlapping data points have been jittered. The graph is based on raw data; the line showing the effect of prey density is fitted using binomial regression

**Figure 3:**
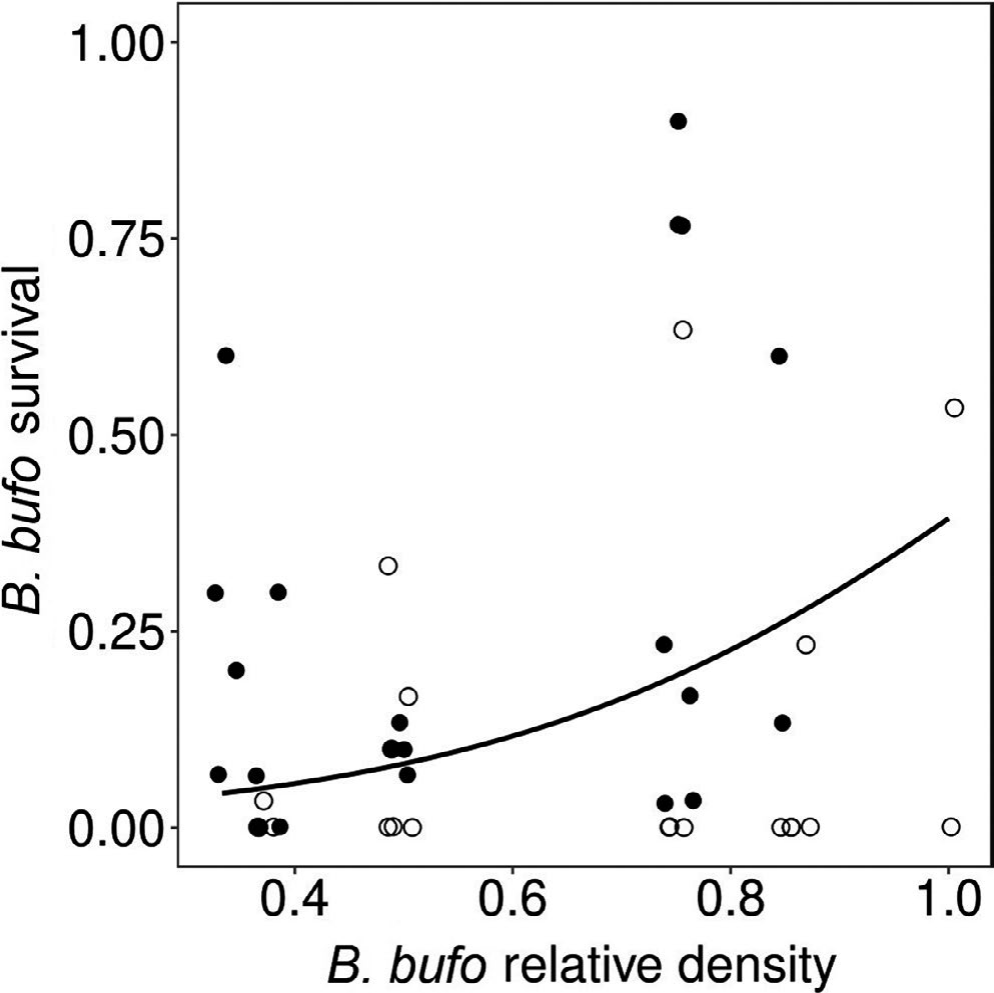
Survival to metamorphosis of moderately chemically defended *B. bufo* tadpoles along the gradient of their initial relative density in two‐species tadpole communities containing undefended, morphologically similar *R. temporaria* tadpoles, in experimental enclosures with a fish predator. Initial relative density is expressed as the proportion of *B. bufo* tadpoles to the total initial number of tadpoles. The initial absolute density of *B. bufo* was fixed across all treatments (30 tadpoles/enclosure). Data points are individual replicates. Circles indicate enclosures with low (empty circles) or high (filled circles) levels of alternative nontadpole food for the predator (fish feed pellets). The overlapping data points have been jittered. The graph was based on raw data; the line showing the effect of the relative density of the chemically defended species was fitted using binomial regression

When *B. bufo* tadpoles were reared in a single‐species prey system, their survival to metamorphosis was positively related to both the availability of alternative nontadpole food and initial tadpole density (Table 2; Figure 2). In the two‐species prey system, *B. bufo* survival was highly positively related to the availability of alternative nontadpole food and to the initial density of the species relative to *R. temporaria* (Table 2; Figure 3). The availability of alternative nontadpole food was decisive for the survival of *R. temporaria* since no tadpoles of this species survived to metamorphosis in the low‐food enclosures. In the high‐food treatment, *R. temporaria* survival increased with the increasing relative proportion of *B. bufo* (*F* = 18.32, *df* = 1, 18.8, *p* < .001; effect size: 16.49 ± SE 3.85).

**Table 2:** GLMM (binomial error structure, logit link) results for the fixed factors affecting *B. bufo* tadpole survival to metamorphosis in the presence of fish

System	Fixed factor	*F*	*df*	*p*	Effect (SE)
Single‐species prey (*B. bufo* *)*	Abundance of alternative food	12.09	1, 12.3	.004	2.787 (0.802)
	Initial absolute density of *B. bufo*	6.08	1, 13.8	.027	0.052 (0.021)
Two‐species prey *B. bufo* with *R. temporaria*)	Abundance of alternative food	10.07	1, 45.0	.003	1.770 (0.558)
	Initial relative density of *B. bufo*	4.40	1, 45.0	.042	2.698 (1.286)

Note

Availability of alternative nontadpole food for fish (low vs. high) was used as a proxy for the hunger state of the predator, and the initial absolute density of *B. bufo* (in single‐species prey system) and the initial relative density of *B. bufo* (in two‐species prey system) were fixed terms. The pond and year were included as random factors. GLMM coefficients are reported with standard errors; for the level of alternative food (categorical factor), the standard errors of differences are presented.

**Figure 4:**
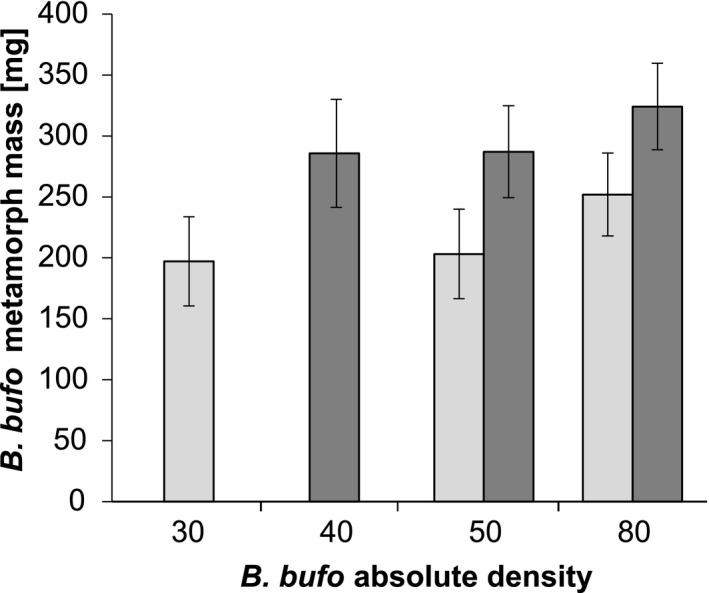
Metamorph mass (mean ± SE) of *B. bufo* (*N* = 436) specimens along the gradient of their initial absolute density in experimental enclosures with common carp *C. carpio*. Lightly‐shaded bars indicate treatments with low levels of alternative (non‐tadpole) food for fish, dark shaded bars indicate treatments with high levels of alternative food. Particular combinations of treatments are not represented because either some enclosures were excluded from the experiment, or very few tadpoles survived to metamorphosis (<3 metamorphs)

**Figure 5:**
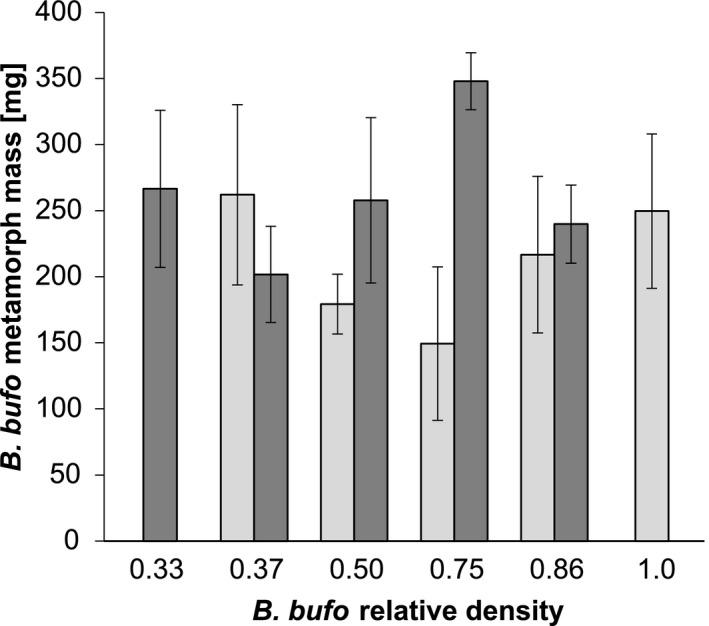
Metamorph mass (mean ± SE) of *B. bufo* (*N* = 166) specimens along the gradient of their initial relative density in experimental tadpole communities with *R. temporaria* under predation pressure from the common carp *C. carpio*. Lightly‐shaded bars indicate treatments with low levels of alternative (non‐tadpole) food for fish, dark shaded bars indicate treatments with high levels of alternative food. Particular combinations of treatments are not represented because either some enclosures were excluded from the experiment, or very few tadpoles survived to metamorphosis (<3 metamorphs)

The *B. bufo* mass at metamorphosis was higher in the high‐food enclosures than in low‐food enclosures (Table 3; Figures 4 and 5). However, neither the effect of the initial density of conspecifics in the single‐species prey system nor the effect of the initial relative density of the *B. bufo* tadpoles in the two‐species prey system was significant (Table 3).

**Table 3:** GLMM (normal error structure, identity link) results for the fixed factors affecting *B. bufo* metamorph mass in the presence of fish

System	Fixed factor	*F*	*df*	*p*	Effect (SE)
Single‐species prey (*B. bufo*)	Abundance of alternative food	12.28	1, 5.5	.015	64.71 (18.46)
	Initial density of *B. bufo*	4.48	1, 9.5	.062	0.80 (0.34)
Two‐species prey (*B. bufo* with *R. temporaria*)	Abundance of alternative food	5.87	1, 11.0	.034	88.53 (36.56)
	Initial relative proportion of *B. bufo*	2.87	1, 14.4	.112	88.93 (52.52)
	Final number of *B. bufo*	0.62	1, 10.1	.450	−1.530 (1.946)

Note

The availability of alternative nontadpole food for the fish (low vs. high) was used as a proxy for the hunger state of the predator, and the initial absolute density of *B. bufo* (in single‐species prey system) and the initial relative density of *B. bufo* and the number of metamorphs (two‐species prey system) were fixed terms. Year and enclosure nested within pond were included as random factors. GLMM coefficients are reported with standard errors; for the level of alternative food (categorical factor), the standard errors of the differences are presented. The interactions between alternative food level and density (or relative proportion) of *B. bufo* were nonsignificant and removed from the analyses (*p* ≥ .40).

## 4 DISCUSSION

We tested the survival of moderately chemically defended aposematic prey following approximately one month of cohabitation with a predator under varying conditions of prey density and predator's physiological state. The advantage of the long duration of the experiment is that it makes it possible for a wide array of learning processes, such as long‐term habituation of predator and prey and predator learning, to influence predator–prey interactions, given that experience‐based responses of this kind will not emerge during short trials aimed at assessing predator and prey behavior (Briggs & Borer, 2005; Turner, 1997). Since the long‐term cumulative effect of predation shapes the survival of prey and drives the evolution of antipredator adaptations, we believe that the results of this study are valid for natural ecosystems, where predation pressure is continuous and intense. However, we acknowledge that mesocosm experiments may extrapolate poorly to complex large‐scale environments. The limited available space and simplified habitat structure in the enclosures may have canceled the effects of species‐specific tadpole antipredator behaviors (e.g., due to the lack of effective refuges); on the other hand, they should not have biased the effects of chemical defenses and density of prey. It should also be noted that the simplified environment of the enclosures did not necessarily translate into very high predation pressure, as the fish used in the experiment are not specialized predators, but slow‐moving omnivores that feed mainly in the bottom sediments (Sibbing, 1988).

In the single‐species prey treatments, we observed increasing survival with increasing absolute density of *B. bufo* tadpoles. This aligns with the theoretical background suggesting that small chemically defended prey should occur at high densities or aggregate to efficiently benefit from their defenses (Sillén‐Tullberg & Leimar, 1988), as well as previous experimental evidence showing that the survival of *B. bufo* tadpoles increases with group size (Watt et al., 1997). Here, we manipulated only the absolute density of the prey and did not directly control the prey aggregation behavior. However, we assumed that in the confined space of the experimental enclosures, the predator was able to simultaneously detect a majority of the prey. Thus, the prey density was functionally equivalent to the prey aggregation as defined in most experimental studies (see Ruxton & Sherratt, 2006). Typically, if the defended prey is present in high densities and/or is aggregated, a lower proportion experience mortality or injury as a result of sampling by the predator (Curley et al., 2015; Riipi et al., 2001). Although the absolute number of sampled individuals may actually increase with prey abundance, the per capita mortality is still expected to decrease due to dilution effect (Rowland et al., 2010; Watt et al., 1997). In the investigated system, tadpoles were subject to predation for a much longer period than in most experiments involving predation trials (e.g., Hatle & Salazar, 2001; Mappes & Alatalo, 1997; Sandre et al., 2010; Sillén‐Tullberg, 1990). Therefore, we assumed that the fish predators had enough time to sample all individuals of the defended prey. Thus, we argue that the dilution effect alone is insufficient to explain the observed pattern. Instead, we suggest that the results were genuinely caused by avoidance learning, that is, the fish learned about the prey defenses after sampling a number of chemically defended tadpoles and refrained from further sampling (“acquisition phase” and “asymptotic phase” of aversive learning, respectively; Skelhorn et al., 2016). The process of learning to avoid defended prey has been demonstrated in fish (Caller & Brown, 2013; Giménez‐Casalduero et al., 1999; Glandt, 1984). Alternatively, the predators may continue to sample the defended prey, but sampling may become less detrimental (i.e., inflicting fewer injuries) to prey with growing predator experience, which correlates with prey density (Kruse & Stone, 1984; Nelson et al., 2010). Another explanation of the observed pattern that does not involve any changes in the foraging strategy of the predators could be that the crowding of *B. bufo* tadpoles drove an increase in their toxin levels in the high‐density treatments, as shown by Bókony et al. (2018). However, the tadpole densities in our enclosures were much lower than in microcosms used by Bókony et al. (2018). Hence, we assume that the potential density‐driven changes in toxin production, even in the high‐density treatments, were too small to explain the observed results. Additionally, the lack of density effects on the metamorph body mass suggested that intraspecific competition in *B. bufo* was low.

In treatments where *B. bufo* tadpoles were raised with undefended *R. temporaria* tadpoles, we observed decreased survival of both species with decreasing relative density of *B. bufo*. This shows that the presence of undefended, roughly similar but nonmimetic heterospecifics can negatively affect the survival of the defended prey. The observed survival pattern contradicts the dilution effect, that is, the increased survival of the defended prey at overall high prey densities. We suspect that the presence of the undefended tadpole‐shaped prey interfered with the avoidance learning strategy of the predator and degraded the protection of the defended tadpoles, as seen in Batesian mimicry systems (cf. Lindström et al., 1997). In general, the predators learn about prey defenses more quickly if the defended prey are easily distinguishable (hence the ubiquity of aposematic signaling) and frequently encountered (Gagliardo & Guilford, 1993; Roper & Wistow, 1986). The presence of undefended yet similar prey in the system weakens the “punishing effect” of attacking defended prey (Lindström et al., 1997; Pfennig et al., 2001). The fish apparently did not refrain from consuming defended tadpoles when they occurred at low relative densities. In turn, high relative density of the defended prey may benefit their undefended neighbors (Mappes et al., 1999), and lead to generalized avoidance of tadpole‐shaped prey, at least when alternative food is abundant (Kaczmarek et al., 2018). It has been demonstrated that some predators use body shape as a complimentary cue to distinguish the defended prey, along with actual warning signals, such as color patterns (Dolenská et al., 2009; Kauppinen & Mappes, 2003; Valkonen et al., 2011). The generalized avoidance (Kaczmarek et al., 2018; Nelson et al., 2010) or attraction (this study) toward tadpole prey suggests that similar mechanisms may also exist in fish. Basically, fish are able to taste and expectorate unpalatable prey (Kasumyan & Sidorov, 2010), resulting in selective consumption of undefended tadpoles (Nelson et al., 2011). However, the generalized attraction toward tadpole‐shaped prey may affect prey handling behavior in fish—fish initially perceiving defended tadpoles as edible often kill them during handling, despite refusing them afterward (Kruse & Stone, 1984; Nomura et al., 2011).

In both experiments, the high availability of alternative nontadpole food for carp significantly increased survival of the *B. bufo* tadpoles. A predator suffering nutrient deficiency is more likely to attack a defended prey item (Hileman et al., 1994; Nonacs, 1985), even if it is already aware of the defenses of the prey (Barnett et al., 2007). Thus, aposematic signaling of defenses, as well as mimicry, appears more likely to evolve in habitats rich in alternative nonmimetic prey that provide easy access to nutrients (Sherratt, 2003). As our results for the two‐species prey system generally complied with the predictions of Batesian mimicry, we could expect the hunger level of the predator and the relative density of the aposematic model species to interact. However, this was not the case, although the main effects were significant. This was presumably because of the dramatic effect of the predator hunger on the survival of both the defended and undefended prey (cf. Sandre et al., 2010).

Although the *B. bufo* survival patterns generally fit our predictions of increased survival with increasing density (both absolute and relative to that of the undefended "mimics"), it must be mentioned that in several enclosures, the survival rates of the *B. bufo* tadpoles were unexpectedly low (i.e., null). As all fish used in the experiments were the same age, close in size, and tadpole‐naïve, as well as similarly fed before entering the experiment, it seems that individual variation in fish personality or ability to metabolize toxins could have contributed to these differences. Fish are known to exhibit varying levels of aggressiveness toward their potential amphibian prey (Winandy & Denoël, 2015). Thus, the individual traits of fish may play an important role in shaping their predation of the defended prey (Nyström & Åbjörnsson, 2000), as has been observed in birds (Bosque et al., 2018; Exnerová et al., 2010, 2015). The abundance of nontadpole food also had a measurable effect on the *B. bufo* metamorph mass; heavier metamorphs emerged from the enclosures where the carp was provided with more fish feed. Fish may exert some indirect positive effects on tadpole and metamorph characteristics, such as by reducing competition (population thinning; Relyea, 2007) and promoting algal growth by nutrient cycling, thus providing extra food resources for the anuran larvae (Benitez‐Mandujano & Flores‐Nava, 1997; Costa & Vonesh, 2013; Kloskowski, 2018); also, we cannot rule out a direct consumption of the fish feed by tadpoles. In our study, metamorph mass was positively but only marginally significantly related to initial *B. bufo* density, indicating that intraspecific competition was not an important factor. The effect of tadpole density on metamorph mass is usually expected to be negative (Goater, 1994; Griffiths & Foster, 1998). However, evidence exists that toad tadpoles may also grow smaller when raised below some threshold density levels, with limited opportunities for social behavior (Wilbur, 1977; Yagi & Green, 2016).

The evolution of aposematism depends on a variety of factors extrinsic to the defended prey, including the state of the predator and the quality of habitat it shares with the prey (Mappes et al., 2005). Our findings showed that under conditions of long‐lasting predation pressure, high densities of moderately defended prey (which were presumably functionally equivalent to, or enhanced by, aggregation behavior) reduced their mortality levels. Intuitively, ample availability of alternative prey for the predator confers survival advantages to the defended prey, as confirmed by improved *B. bufo* survival in the treatments where the predator received additional nontadpole food. From the perspective of the evolution of aposematism, it is logical that the presence of alternative prey in general should promote the spread of aposematism (Sherratt, 2003). However, our results showed that the beneficial role of alternative prey may be at least partly reversed when a predator confuses the defended prey with the harmless prey. The resemblance between the defended and undefended prey in our study system could be treated as imperfect and accidental, with the undefended species lacking selection‐driven imitation of the aposematic signal. Nevertheless, it inflicted substantial survival costs on the defended species. Our data indicated that if predators tend to broadly generalize prey characteristics, the occurrence of similarly shaped, undefended prey in the system may affect the initial stages of the evolution of aposematism through mimicry‐like effects. If these effects are not offset by high densities, strong defenses or unmistakable aposematic signal of the model species, facilitating prey discrimination by the predators (cf. Gamberale‐Stille, 2001; Lindström et al., 2001; Skelhorn & Rowe, 2006), the spread and maintenance of the aposematic traits may be inhibited.

## CONFLICT OF INTEREST

The authors declare no conflict of interests.

## AUTHOR CONTRIBUTION

**Jan Marek Kaczmarek:** Conceptualization (equal); Data curation (lead); Formal analysis (equal); Funding acquisition (equal); Investigation (equal); Methodology (equal); Writing‐original draft (lead); Writing‐review & editing (equal). **Mikołaj Kaczmarski:** Investigation (equal); Visualization (equal); Writing‐review & editing (supporting). **Jan Mazurkiewicz:** Funding acquisition (supporting); Investigation (equal); Resources (equal); Writing‐review & editing (supporting). **Janusz Kloskowski:** Conceptualization (equal); Formal analysis (lead); Investigation (equal); Methodology (equal); Supervision (lead); Validation (equal); Writing‐original draft (equal); Writing‐review & editing (equal).

## Data Availability

The data used for analyses provided in the article are available at Dryad under https://doi.org/10.5061/dryad.w9ghx3fn2
